# Anti-Inflammatory Effects on Periodontal Tissue and Antibacterial Effects on Oral Bacteria of Chlorogenic Acid

**DOI:** 10.3390/dj14020125

**Published:** 2026-02-22

**Authors:** Yuya Suzuki, Kosuke Maruyama, Masato Mikami, Soh Sato

**Affiliations:** 1 Field of Advanced Conservative Dentistry and Periodontology, Periodontology, Course of Clinical Science, The Nippon Dental University Graduate School of Life Dentistry at Niigata, 1-8 Hamaura-cho, Chuo-ku, Niigata 951-8580, Japan; yssuzuki@ngt.ndu.ac.jp; 2 Department of Microbiology, The Nippon Dental University School of Life Dentistry at Niigata, 1-8 Hamaura-cho, Chuo-ku, Niigata 951-8580, Japan; 3 Department of Periodontology, The Nippon Dental University School of Life Dentistry at Niigata, 1-8 Hamaura-cho, Chuo-ku, Niigata 951-8580, Japan

**Keywords:** chlorogenic acid, periodontal disease, chemical plaque control, *Streptococcus mutans*, *Aggregatibacter actinomycetemcomitans*, *Porphyromonas gingivalis*, *Fusobacterium nucleatum*

## Abstract

**Objectives**: Combining mechanical plaque control, the physical removal of oral biofilm, with chemical plaque control, the use of agents to inhibit biofilm formation, is effective in preventing periodontal disease. Chlorogenic acid (CGA) found in coffee beans has medicinal effects, such as anti-inflammatory and antibacterial properties. Periodontal pathogens are difficult to reach in certain areas with traditional self-care tools, such as toothbrushes. Additionally, the viscous biofilm is difficult to remove using mechanical plaque control alone. Therefore, this study aimed to evaluate the efficacy of CGA in chemical plaque control. **Methods**: The mRNA and protein expression of inflammatory cytokines in lipopolysaccharide (LPS)-stimulated human gingival fibroblasts (HGFs) and human periodontal ligament fibroblasts (HPDLs) in the presence of CGA were analyzed using reverse transcription-qPCR and enzyme-linked immunosorbent assay. Additionally, the proliferation levels of oral bacteria in the presence of CGA were evaluated. **Results**: CGA suppressed mRNA and protein expression levels of the inflammatory cytokines, interleukin (IL)-1β and IL-8, in HGFs and HPDLs stimulated with *Porphyromonas gingivalis* LPS. Furthermore, CGA inhibited bacterial proliferation of *Streptococcus mutans*, *Aggregatibacter actinomycetemcomitans*, *P. gingivalis*, and *Fusobacterium nucleatum*. **Conclusions**: This study demonstrated that CGA exhibits anti-inflammatory effects on gingiva and periodontal ligaments, and antibacterial effects against oral bacteria. These results indicate the potential application of CGA in chemical plaque control and suggest its use in preventing periodontal disease progression.

## 1. Introduction

Periodontal disease is a chronic inflammatory disease that destroys periodontal tissues [1]. The progression of this disease causes tooth loss, leading to poor nutritional status due to reduced masticatory function, thereby increasing mortality rates and the risk of developing lifestyle-related diseases. Therefore, preventing periodontal disease is crucial for reducing the risk of systemic diseases [2,3,4].

The biofilm adhering to tooth surfaces, composed of oral bacteria, is called dental plaque. It is a highly viscous structure that is difficult to completely remove physically, making it prone to residual presence and a cause of periodontal disease onset [5]. As the biofilm matures, its constituent bacteria change. Early-stage biofilm is primarily composed of Gram-positive bacteria, such as *Streptococcus mutans*, but later-stage biofilm contains abundant periodontal pathogens [5,6]. Lipopolysaccharide (LPS), derived from periodontal pathogens (Gram-negative rods), is a key pathogenic factor in periodontal disease onset, involved in inflammatory cytokine production, bone resorption, and exacerbation of inflammation [7]. LPS binds to Toll-like receptor 4 (TLR4) on the surface of human gingival fibroblasts (HGFs), which are abundant in gingival connective tissue and contributes to the release of inflammatory cytokines [8]. The inflammatory cytokines, interleukin (IL)-1β and IL-8, play central roles in the progression of periodontal disease. IL-1β is a major factor inducing the production of inflammatory mediators from immune and tissue cells, promoting osteoclast differentiation and contributing to bone destruction [9]. IL-8 is a potent chemotactic factor for neutrophils, promoting their local activation. Persistent neutrophil infiltration causes tissue destruction [10]. Therefore, suppressing IL-1β and IL-8 expression levels may prevent periodontal disease progression.

Removing biofilm is effective for preventing periodontal disease progression, making mechanical plaque control crucial [11]. Periodontal pathogens are anaerobic bacteria that thrive in the oxygen-deprived gingival sulcus, making it difficult for self-care tools, such as toothbrushes, to reach this area [12]. The viscous biofilm strongly adheres to the tooth surface, making it difficult to remove using mechanical plaque control alone [5,13]. Therefore, chemical plaque control, which uses drugs to suppress biofilm formation on the tooth surface, is effective as an adjunctive therapy [14]. The combination of mechanical and chemical plaque control is important in preventing the progression of periodontal disease.

Chlorogenic acid (CGA) is a polyphenol abundant in coffee beans, eggplants, potatoes, and apples, and a general term for nine types of ester compounds derived from cinnamic and quinic acid [15]. The most abundant of the nine types is 5-caffeoylquinic acid [16]. CGA has anti-inflammatory, antibacterial, antioxidant, cardioprotective, and hepatoprotective effects [17]. In the dental field, CGA exhibits antibacterial activity by inhibiting the growth of *Porphyromonas gingivalis* and suppresses the expression levels of inflammatory cytokines in LPS-stimulated HGFs [18]. Previous reports on CGA in dentistry involved a few bacterial species and periodontal tissue cells have only been reported for HGFs. However, to the best of our knowledge, this is the first study to examine the effects of CGA as a chemical plaque control on *S. mutans* and *Aggregatibacter actinomycetemcomitans*, and inflammatory cytokine expression in human periodontal ligament fibroblasts (HPDLs).

## 2. Materials and Methods

### 2.1. Isolation of HGFs and HPDLs

Gingival tissue was obtained from the gingiva and periodontal ligament attached to teeth extracted from healthy, non-smoking patients (*n* = 8, mean age 37.6 ± 11.4 years) who visited Niigata Hospital, Nippon Dental University, Niigata, Japan. Regarding the use of extracted teeth, prior consent was obtained through a consent form. Tissues were prepared by mincing the gingival tissue into approximately 1 mm^3^ sections using the method described by Sato et al. [19]. The tissue sections were placed in a 35 mm culture dish (Corning, Newark, NJ, USA) containing Dulbecco’s Modified Eagle’s Medium (DMEM) Nutrient Mixture F-12 (Thermo Fisher Scientific, Waltham, MA, USA) supplemented with 15% fetal bovine serum (FBS; JR Scientific, Woodland, CA, USA), 100 U/mL of penicillin (Invitrogen, Carlsbad, CA, USA), 100 µg/mL of streptomycin (Thermo Fisher Scientific), and 250 µg/mL of amphotericin B (Invitrogen). The dishes were incubated in an incubator at 37 °C with 5% CO_2_ and 95% air. This study was approved by the Ethics Review Committee of the Niigata School of Life Dentistry, Nippon Dental University (approval number: ECNG-R-391, Date of Recognition: 2 March 2023). The study was conducted in accordance with the Declaration of Helsinki principles in the methodology section.

### 2.2. Culture Conditions

HGFs and HPDLs were cultured were cultured in DMEM containing 15% FBS supplemented with 100 μL of *P. gingivalis* LPS (InvivoGen, San Diego, CA, USA) at a concentration of 1 µg/mL. The primary form of chlorogenic acid (Sigma-Aldrich, St. Louis, MO, USA, ≥95% titration) was dissolved in DMSO (FUJIFILM Wako Pure Chemical Corporation, Osaka, Japan) and adjusted to concentrations of 0.5, 5, 50, 100, 200, and 500 µM in DMEM containing 15% FBS. Two groups were established: one group with HGFs and HPDLs cultured in medium supplemented with CGA and LPS (CGA + LPS group), and another group with HGFs and HPDLs in medium supplemented with CGA only (CGA group). HGFs and HPDLs cultured in medium containing only LPS (LPS group) served as the negative control group, whereas HGF and HPDL cultured in standard medium without CGA or LPS served as the positive control group (Control group).

### 2.3. Analysis of the CGA Effects on Cell Proliferation

HGFs and HPDLs were prepared at a concentration of 1.5 × 10^3^ cells/mL in DMEM containing 15% FBS and seeded at 100 μL per well in a 96-well plate (Corning). After 24 h of seeding, the medium was replaced in each experimental group and changed every 24 h. CGA was prepared by dissolving (≥95% titration; Sigma-Aldrich) in DMSO (FUJIFILM Wako Pure Chemical Corporation) to a final concentration of 50 mM. The 50 mM CGA solution was diluted to various concentrations using the culture medium. The LPS group contained LPS-PG (standard) (Invivogen), adjusted to 1 µg/mL using the culture medium. The DMSO group was adjusted to contain the same volume of DMSO as the highest CGA concentration. As previously described by Sato et al. [19], on days 1, 2, 4, 6, and 8 of culture, mitochondrial reduction staining was performed using the alamarBlue Cell Viability Assay (Thermo Fisher Scientific), and fluorescence was measured using a microplate reader (POWERSCAN MX, DS Pharma Biomedical Co. Ltd., Osaka, Japan). A standard curve was created based on cell count and fluorescence intensity, and the data were expressed as cell numbers. Cell proliferation assays were conducted in all experimental groups.

### 2.4. Analysis of IL-1β and IL-8 mRNA Expression Levels

To analyze the mRNA expression levels of *IL-1β* and *IL-8*, we performed RT-qPCR. HGF and HPDL were prepared at a concentration of 1.0 × 10^5^ cells/mL in DMEM containing 15% FBS. Cells (100 μL) were seeded into a 60 mm dish (Corning) and incubated in DMEM for 2 days. CGA was prepared by dissolving (Sigma-Aldrich) in DMSO (FUJIFILM Wako Pure Chemical Corporation) to a final concentration of 50 mM. The 50 mM CGA solution was diluted to various concentrations using culture medium. The LPS group contained LPS-PG (standard) (Invivogen) adjusted to 1 µg/mL using culture medium. The DMSO group was adjusted to contain the same volume of DMSO as the highest CGA concentration. In accordance with Suzuki et al. [20], cells were stimulated with *P. gingivalis* LPS for 6 h, followed by exposure to CGA for 2 h. Total RNA was extracted using NucleoSpin RNA (Takara Bio Inc, Shiga, Japan) according to the manufacturer’s instructions. Complementary DNA (cDNA) were synthesized from 1 µg of total RNA using a high-capacity cDNA reverse transcription kit (Thermo Fisher Scientific). IL-1β and IL-8 expression levels were analyzed using the StepOnePlus Real-time PCR System (Thermo Fisher Scientific). cDNA (50 ng) was mixed with 0.2 µM forward and reverse primers (Table 1; Fasmac, Kanagawa, Japan) in 25 µL Power SYBR Green PCR Master Mix (Thermo Fisher Scientific). PCR cycling conditions were as follows: 10 min at 95 °C for denaturation, followed by 40 cycles of 15 s at 95 °C for denaturation, and 1 min at 60 °C for annealing/extension. Data were relatively quantified using the comparative cycle threshold (ΔΔCt) method with StepOne Software Version 2.2 (Thermo Fisher Scientific).

### 2.5. Analysis of IL-1β and IL-8 Protein Expression

To analyze the protein expression of IL-1β and IL-8, an enzyme-linked immunosorbent assay (ELISA) was performed. HGFs and HPDLs were prepared at a concentration of 1.0 × 10^5^ cells/mL in DMEM containing 15% FBS. The cell suspension (3 mL) was seeded into 60 mm dishes (Corning) and incubated in DMEM for 2 days. CGA was prepared by dissolving (Sigma-Aldrich) in DMSO (FUJI-FILM Wako Pure Chemical Corporation) to a final concentration of 50 mM. The 50 mM CGA solution was diluted to various concentrations using culture medium. The LPS group used LPS-PG (standard) (Invivogen), adjusted to 1 µg/mL using medium. The DMSO group was adjusted to contain the same volume of DMSO as the highest CGA concentration. Cells were exposed to *P. gingivalis* LPS for 6 h, followed by exposure to CGA for 48 h. IL-1β released into the culture medium from HGFs and HPDLs was quantified using the Human IL-1β Sandwich ELISA Kit (Proteintech, Rosemont, IL, USA) and IL-8 was quantified using the Human IL-8 Sandwich ELISA Kit (Proteintech) according to the manufacturer’s protocol.

### 2.6. Bacterial Strains and Culture Conditions

The following bacterial strains were used: *S. mutans* OMZ175, *A. actinomycetemcomitans* ATCC 43718, *Fusobacterium nucleatum* ATCC 25586, and *P. gingivalis* 381. *S. mutans* and *A. actinomycetemcomitans* were aerobically cultured at 37 °C in tryptic soy broth (TSB) medium (Becton Dickinson, Franklin Lakes, NJ, USA) in an incubator containing 10% CO_2_ and 90% air. *P. gingivalis* and *F. nucleatum* were cultured in Gifu anaerobic medium (GAM; Shimadzu Diagnostics Corporation, Tokyo, Japan) supplemented with 5 µg/mL of hemin (FUJIFILM Wako Pure Chemical Corporation) and 0.5 µg/mL of vitamin K (FUJIFILM Wako Pure Chemical Corporation), and anaerobically cultured at 37 °C in an incubator containing 70% N_2_, 20% CO_2_, and 10% H_2_. CGA was dissolved in DMSO (FUJIFILM Wako Pure Chemical Corporation), and TSB medium and GAM were used to adjust the CGA concentration to 3.125, 6.25, 12.5, 25, and 50 mM. The CGA and Control groups were used as controls.

### 2.7. Analysis of CGA Effects on Bacterial Proliferation

*S. mutans* and *A. actinomycetemcomitans* were cultured on TSB agar plates for 48 h, then cultured in TSB for 48 h. *P. gingivalis* and *F. nucleatum* were cultured on GAM agar plates for 48 h, cultured in GAM for 48 h. *S. mutans* and *A. actinomycetemcomitans* were adjusted to 1.0 × 10^6^ CFU/mL in TSB, whereas *P. gingivalis* and *F. nucleatum* were adjusted in GAM. Each was inoculated into 3 mL of their respective medium and cultured for 48 h. CGA was dissolved in DMSO (FUJIFILM Wako Pure Chemical Corporation) to prepare a 100 mM CGA stock solution. This stock solution was then diluted to various concentrations in the culture medium. The DMSO control group was prepared by adding an equal volume of DMSO to the highest CGA concentration used. Every 6 h, the cultures were vortexed, and 100 µL of samples were added to a 96-well plate (Corning). In accordance with Wang et al. [21], absorbance was measured at 570 nm using a microplate reader (POWERSCAN MX, DS Pharma Biomedical Co. Ltd.).

### 2.8. Statistical Analysis

The Kolmogorov–Smirnov test was used to confirm that the data did not follow a normal distribution. Therefore, the Kruskal–Wallis test was used for statistical analysis and Steel–Dwass test was used for multiple comparisons. Statistical software BellCurve for Excel, version 4.05 (Social Survey Research Information Co. Ltd., Tokyo, Japan) was used. A *p*-value less than 0.05 was considered statistically significant. A standard curve was created based on bacterial colony counts and absorbance values, and the data were expressed as bacterial counts (CFU/mL). Cell proliferation assays were performed in all experimental groups.

## 3. Results

### 3.1. Cell Proliferation

**Figure 1:**
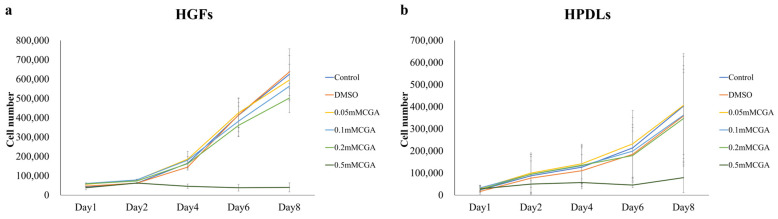
Cell proliferation of HGFs and HPDLs cultured with various concentrations of CGA. (**a**) Effect of CGA on HGFs. (**b**) Effects of CGA on HPDL. Cell numbers for CGA and control groups. The control used cells cultured in medium alone. The data presented are the mean ± standard deviation. The Kruskal–Wallis test was used for statistical analysis and the Steel–Dwass test was used for multiple comparisons. *n* = 8. CGA, chlorogenic acid; HPDL, human periodontal ligament fibroblast; HGF, human gingival fibroblast.

**Figure 2:**
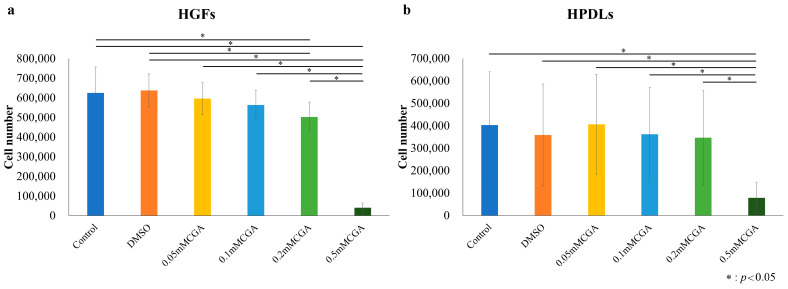
Cell proliferation on day 8 of HGFs and HPDLs cultured with various concentrations of CGA. (**a**) Cell numbers on day 8 of HGFs for CGA and control groups. (**b**) Cell numbers on day 8 of HPDLs for CGA and control groups. The control used cells cultured in medium alone. Cell numbers at day 8 for CGA and control groups. The data presented are the mean ± standard deviation. The Kruskal–Wallis test was used for statistical analysis and the Steel–Dwass test was used for multiple comparisons. * *p* < 0.05, *n* = 8. CGA, chlorogenic acid; HPDL, human periodontal ligament fibroblast; HGF, human gingival fibroblast.

**Figure 3:**
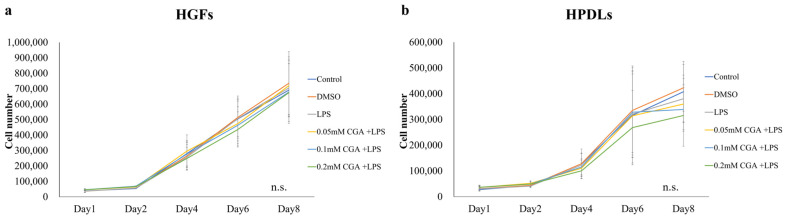
Cell proliferation of *P. gingivalis* LPS-stimulated HGFs and HPDLs calculated with various CGA concentrations. (**a**) Cell proliferation of *P. gingivalis* LPS-stimulated HGFs calculated with various concentrations of CGA. Effects of CGA and LPS on HGFs. (**b**) Cell proliferation of *P. gingivalis* LPS-stimulated HPDLs calculated with various concentrations of CGA. The control used cells cultured in medium alone. The data presented are the mean ± standard deviation. The Kruskal–Wallis test was used for statistical analysis and Steel–Dwass test was used for multiple comparisons. n.s.: not significant, *n* = 8. CGA, chlorogenic acid; HPDL, human periodontal ligament fibroblast; HGF, human gingival fibroblast; LPS, lipopolysaccharide.

HGFs and HPDLs exhibited cell proliferation over time, continuing until day 8 (Figure 1a,b). Significant proliferation inhibition was observed in HGFs on day 8 between control and 0.05 mM CGA, 0.1 mM CGA, 0.2 mM CGA, and 0.5 mM CGA groups. Compared with the control group, a significant suppression of proliferation was observed at 0.2 mM CGA (Figure 2a). Compared to the DMSO group, a significant inhibition of cell proliferation was observed at 0.5 mM CGA (Figure 2a). For HPDLs, significant suppression of proliferation was observed on day 8 between control, 0.05 mM CGA, 0.1 mM CGA, 0.02 mM CGA, and 0.5 mM CGA groups (Figure 2b). Compared to DMSO, a significant inhibition of cell proliferation was observed at 0.5 mM (Figure 2b). When HGFs and HPDLs were stimulated with 1.0 mg/mL of *P. gingivalis* LPS, cell proliferation was observed over time and continued until day 8. No significant differences were observed between conditions for either HGFs or HPDLs at any time point (Figure 3).

### 3.2. IL-1β and IL-8 of mRNA Expression Levels by CGA

**Figure 4:**
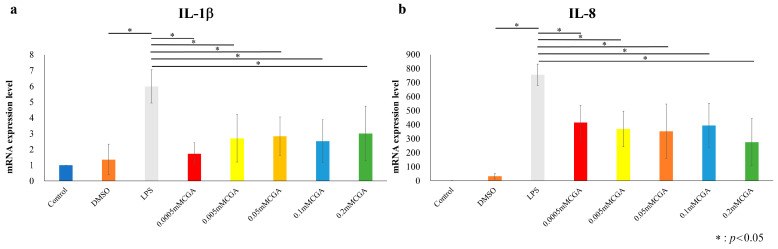
CGA-induced IL-1β and IL-8 of mRNA expression levels in HGFs. mRNA expression levels following a 6 h stimulation with 1 µg/mL of *P. gingivalis* LPS, followed by incubation with various CGA concentrations for 2 h. mRNA expression levels in the experimental groups containing *P. gingivalis* LPS (negative control) are shown as relative values to the mRNA expression levels in the control group (positive control). (**a**) mRNA expression levels of *IL-1β* in HGFs. (**b**) mRNA expression levels of IL-8 in HGFs. The control used cells cultured in medium alone. The data presented are the mean ± standard deviation of duplicate per sample. The Kruskal–Wallis test was used for statistical analysis and the Steel–Dwass test was used for multiple comparisons. * *p* < 0.05, *n* = 8. CGA, chlorogenic acid; HGF, human gingival fibroblast; LPS, lipopolysaccharide.

**Figure 5:**
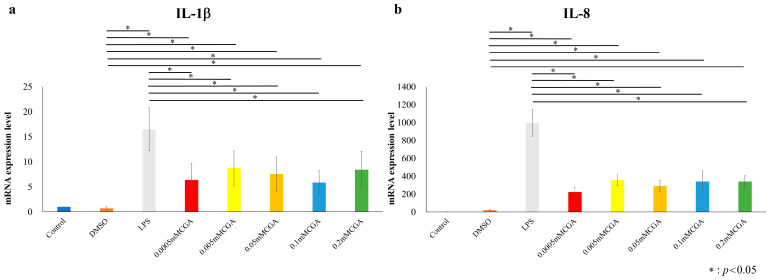
CGA-induced IL-1β and IL-8 of mRNA expression levels in HPDLs. mRNA expression levels following a 6 h stimulation with 1 µg/mL of *P. gingivalis*-LPS, followed by incubation in various CGA concentration for 2 h. mRNA expression levels in the experimental groups containing *P. gingivalis* LPS (negative control) are shown as relative values compared to the mRNA expression levels in the control group (positive control). (**a**) mRNA expression levels of IL-1β in HPDLs. (**b**) mRNA expression levels of IL-8 in HPDLs. The control used cells cultured in medium alone. The data presented are the mean ± standard deviation of duplicate per sample. The Kruskal–Wallis test was used for statistical analysis and the Steel–Dwass test was used for multiple comparisons. * *p* < 0.05, *n* = 8. CGA, chlorogenic acid; HPDL, human periodontal ligament fibroblast; LPS, lipopolysaccharide.

**Figure 6:**
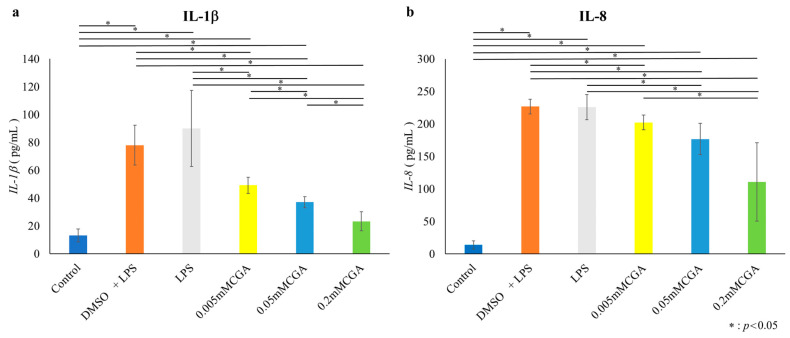
Protein expression of IL-1β and IL-8 from HGFs. Protein expression following a 6 h stimulation with 1 µg/mL of *P. gingivalis* LPS, followed by a 48 h incubation with various CGA concentrations. (**a**) Protein expression of IL-1β in HGFs. (**b**) Protein expression of IL-8 in HGFs. The control used cells cultured in medium alone. The data presented are the mean ± standard deviation of duplicate per sample. The Kruskal–Wallis test was used for statistical analysis and the Steel–Dwass test was used for multiple comparisons. * *p* < 0.05, *n* = 8. CGA, chlorogenic acid; HGF, human gingival fibroblast; LPS, lipopolysaccharide.

After stimulating HGFs and HPDLs with 1 µg/mL of *P. gingivalis*-LPS for 6 h, mRNA expression levels were examined following a 2-h incubation with CGA. (Figure 4 and Figure 5). In HGFs, both *IL-1β* and *IL-8* mRNA expressions were significantly suppressed at CGA concentrations of 0.0005, 0.005, 0.05, 0.1, 0.2 mM compared with those in the LPS group (Figure 4). In HPDLs, both *IL-1β* and *IL-8* mRNA expression levels were significantly suppressed at CGA concentrations of 0.0005, 0.005, 0.05, 0.1, and 0.2 mM compared with those in the LPS group (Figure 6).

### 3.3. CGA-Induced IL-1β and IL-8 Protein Expression

**Figure 7:**
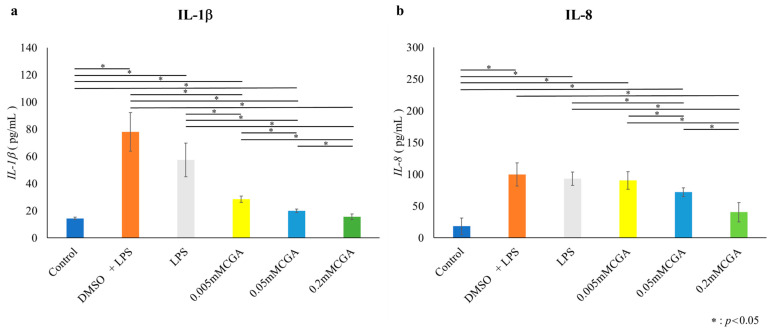
Protein expression of IL-1β and IL-8 from HPDLs. Protein expression following a 6 h stimulation with 1.0 µg/mL of *P. gingivalis* LPS, followed by a 48 h incubation with CGA (0.5, 5, 50, 100, and 200 µM). (**a**) Protein expression of IL-1β in HPDLs. (**b**) Protein expression of IL-8 in HPDLs. The data presented are the mean ± standard deviation of duplicate per sample. The control used cells cultured in medium alone. The Kruskal–Wallis test was used for statistical analysis and the Steel–Dwass test was used for multiple comparisons. * *p* < 0.05, *n* = 8. CGA, chlorogenic acid; HPDL, human periodontal ligament fibroblast; LPS, lipopolysaccharide.

In HGFs, both IL-1β and IL-8 protein expression was significantly suppressed at CGA concentrations of 0.005, 0.05, and 0.2 mM compared with those in the LPS group (Figure 6). In HPDLs, IL-1β and IL-8 protein expression was significantly suppressed at CGA concentrations of 0.005, 0.05, and 0.2 mM compared with those in the LPS group (Figure 7).

### 3.4. Bacterial Proliferation

**Figure 8:**
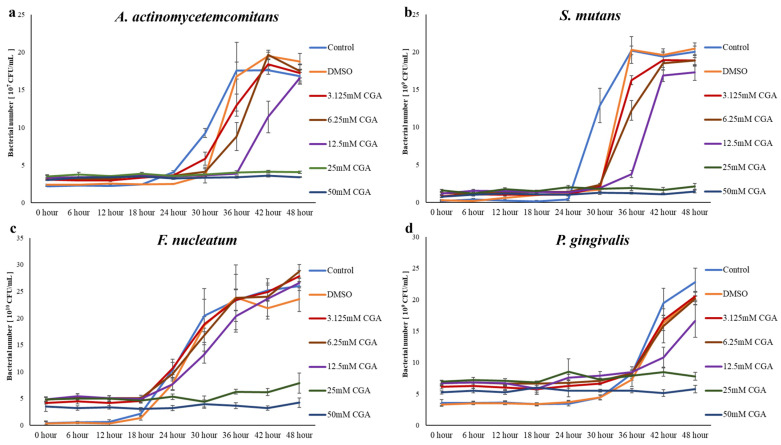
Bacterial proliferation incubated with CGA. (**a**) Effect of CGA on *A. actinomycetemcomitans*. (**b**) Effects of CGA on *S. mutans*. (**c**) Effects of CGA on *F. nucleatum*. (**d**) Effects of CGA on *P. gingivalis*. The control used cells cultured in medium alone. The data presented are the mean ± standard deviation of duplicate per sample. *n* = 8. CGA, chlorogenic acid.

**Figure 9:**
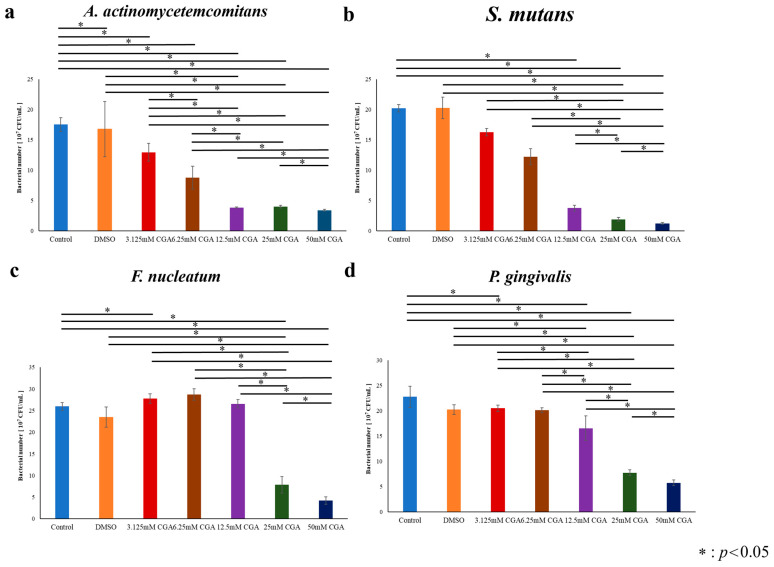
Bacterial proliferation on day 8 incubated with CGA. (**a**) Bacterial number of *A. actinomycetemcomitans* in CGA and control groups at day 8. (**b**) Bacterial number of *S. mutans* in CGA and control groups at day 8. (**c**) Bacterial number of *F. nucleatum* in CGA and control groups at day 8. (**d**) Bacterial number of *P. gingivalis* in CGA and control groups at day 8. Data were converted to CFU after measuring absorbance at 570 nm. The control used cells cultured in medium alone. The data presented are the mean ± standard deviation of duplicate per sample. The Kruskal–Wallis test was used for statistical analysis and the Steel–Dwass test was used for multiple comparisons. * *p* < 0.05, *n* = 8. CGA, chlorogenic acid.

Proliferation of bacteria for 48 h with various concentrations of CGA were evaluated (Figure 8 and Figure 9). *A. actinomycetemcomitans* and *S. mutans* plateaued at 36 h (Figure 8a,b). *F. nucleatum* and *P. gingivalis* plateaued at 48 h (Figure 8c,d). *A. actinomycetemcomitans* proliferation was significantly inhibited at CGA concentrations more than 3.125 mM compared with those in the Control group (Figure 9a). *S. mutans* proliferation was significantly inhibited in the CGA-added groups compared with those in the Control group (Figure 9b). *F. nucleatum* proliferation was significantly inhibited in the 25 and 50 mM CGA groups compared with those in the Control group (Figure 9c). *P. gingivalis* proliferation was significantly inhibited in the 12.5-, 25-, and 50-mM CGA groups compared with that of the Control group (Figure 9d).

## 4. Discussion

In this study, cell proliferation was significantly inhibited in the CGA 500 µM group compared with that in the HGF and HPDL Control groups on day 8 of cell culture. Park et al. reported that exposure of HGFs to CGA at 50 mM did not affect cell viability after 24 h [18]. In the HGFs and HPDLs used in this study, a CGA concentration of 500 µM had a significant impact on cell proliferation and was excluded from subsequent experiments. In HGFs, cell proliferation was significantly inhibited in the CGA 200 µM and Control groups, but no significant difference was found between the CGA 50 and 100 µM groups. Based on these findings, CGA concentrations of 200 µM or lower were used for HGFs and HPDLs in this study. Furthermore, in the HGF and HPDL used in this study, no significant differences in cell proliferation were observed between the Control, LPS, and LPS + CGA 50 µM, CGA 100 µM, or CGA 200 µM groups when CGA was added to *P. gingivalis* LPS-stimulated HGFs and HPDLs. Therefore, when CGA- *P. gingivalis* LPS and CGA are added to HGFs and HPDLs, the effect on cell proliferation is considered to be minimal.

HGFs and HPDLs are the main components of human periodontal connective tissue. When LPS from periodontal pathogens, such as *P. gingivalis*, binds to TLR4 on the cell membrane, TLR4 undergoes a structural change, initiating a signal transduction cascade [22]. Consequently, DNA undergoes transcription and translation, leading to the production and release of inflammatory cytokines, such as IL-1β and IL-8, resulting in gingival inflammation [23]. Periodontal disease is triggered by the release of inflammatory cytokines induced by LPS, exacerbating the destruction of local tissues, including epithelium, connective tissue, and gingival tissue [23]. IL-1β is a potent inflammatory cytokine that activates inflammatory signaling pathways, such as NF-κB and MAPK, inducing inflammation-related genes, such as *IL-8* [24,25]. IL-8 induced by IL-1β promotes neutrophil migration and activation, contributing to the chronicity of periodontal disease [10,26]. In this study, mRNA and protein expression of *IL-1β* and *IL-8* were significantly suppressed in the group treated with LPS + CGA compared with those in the group treated with LPS alone. In the protein expression analysis, IL-1β expression was suppressed in all CGA-treated groups, whereas IL-8 expression was not suppressed at 5 µM of CGA. Liang et al. reported that in human colon adenocarcinoma (Caco-2) cells stimulated with interferon-γ and phorbol myristate acetate, IL-8 protein expression was significantly lower in the 2 mM CGA group than in the 0.2 mM CGA group [27]. A certain CGA concentration is necessary to suppress IL-8 expression. Given its suppression of IL-1β and IL-8 expression, CGA possesses anti-inflammatory effects.

Bacteria are a major cause of periodontal disease [28]. Oral bacteria form biofilms, structures composed of aggregates of microorganisms and substances produced by these microorganisms [5]. Gram-positive facultative anaerobes, such as *Streptococcus sanguinis* and *S. mutans* adhere to tooth surfaces early on, forming initial biofilms [5,6]. Although the pathogenicity of these initial biofilms is low, as they mature, oxygen-depleted environments emerge. Gram-negative anaerobic bacteria become dominant, and the proportion of periodontal pathogens increases [29]. Among mid-stage adherent bacteria, *F. nucleatum*, one of the periodontal pathogens, appears [29]. *F. nucleatum* acts as a bridge bacteria, facilitating the physical and chemical bonding of late-stage adherent bacteria with high pathogenicity, such as *P. gingivalis*, and early-stage adherent bacteria. It acts as a bridge bacteria promoting co-aggregation through physical and chemical bonding, thereby maturing the biofilm to allow attachment of late-stage pathogenic bacteria [29,30]. Late-adherent bacteria possess high pathogenicity, secreting LPS and proteases that induce inflammation and tissue destruction, leading to the onset and progression of periodontal disease [31]. In this study, based on bacterial growth results, CGA exhibited antimicrobial activity against *S. mutans*, *A. actinomycetemcomitans*, *P. gingivalis*, and *F. nucleatum*. Furthermore, by inhibiting the growth of *F. nucleatum*, a bridge bacterium, CGA can prevent the maturation of biofilms and thus prevent the progression of periodontal disease.

Based on the results of this study, CGA possesses anti-inflammatory and antibacterial effects. Moreover, by inhibiting the growth of *F. nucleatum*, CGA was confirmed to prevent biofilm maturation and is applicable for chemical plaque control. This study used cells and bacteria for investigation; however, cells are protected by epithelial structures and bacteria form biofilms, which may differ from the actual oral environment. Further investigation is necessary for the clinical application of CGA.

The results of this study indicate that the CGA concentration affecting cells significantly differs from that affecting bacteria. The CGA concentration in beverages, such as coffee, is approximately 48 mM [32]; therefore, the method used in this study likely caused a strong effect on cells. Furthermore, if used in mouthwash, CGA residue in the oral cavity is expected to be short-lived [33]. HGFs and HPDLs exist within connective tissue and are covered by the epithelial layer, suggesting minimal cellular invasion [34]. When considering CGA as a mouthwash ingredient, evaluating its intraoral action time is necessary. However, the methodology used in this study has limitations. Future research on animal models is required to investigate intraoral activity time of CGA. Furthermore, to clarify direct effects of CGA on bacterial cells, we conducted an evaluation using planktonic cells. Biofilms exhibit complex mechanisms involving physical barriers from the extracellular matrix and metabolic changes. Given the vast diversity and composition of recent biofilm types, elucidating the effects on individual bacteria is considered essential for future studies on the impact of biofilms. This study served as a model case for examining the effects of CGA on bacteria. However, further investigation into the effects of CGA on biofilms is necessary.

## 5. Conclusions

This study demonstrated that CGA exhibits anti-inflammatory effects on the gingiva and periodontal ligament, as well as antibacterial effects against oral bacteria. These findings indicate that CGA can be used for chemical plaque control and suggest its potential use in preventing the progression of periodontal disease. Further investigation is necessary for the clinical application of CGA.

## Figures and Tables

**Table 1:** Primers used in RT-qPCR.

Gene		Primer Sequence 5′ → 3′
*GAPDH*	Forward	GCTCCCTCTTTCTTTGCAGC
	Reverse	CATGAGTCCTTCCACGATACCA
*IL-1β*	Forward	CCAGGGACAGGTATGGAGCA
	Reverse	TTCAACACGCAGGACAGGTACAG
*IL-8*	Forward	GAACCATCTCACTGTGTGTAAA
	Reverse	CACTCCTTGGCAAAACTG

## Data Availability

The datasets presented in this article are not readily available because the data are part of an ongoing study. Requests to access the datasets should be directed to Yuya Suzuki.

## Abbreviations

The following abbreviations are used in this manuscript:

cDNA	Complementary DNA
CFU	Colony forming unit
CGA	Chlorogenic acid
DMEM	Dulbecco’s modified eagle’s medium
ELISA	Enzyme-linked immunosorbent assay
FBS	Fetal bovine serum
GAM	Gifu anerobic medium
HGF	Human gingival fibroblast
HPDL	Human periodontal ligament fibroblast
IL	Interleukin
LPS	Lipopolysaccharide
TSB	Tryptic soy broth
